# London University Course

**Published:** 1917-09-08

**Authors:** 


### LONDON UNIVERSITY COURSE.

The University confers the degrees of Bachelor of Medicine and Bachelor of Surgery (M.B., B.S.), Doctor of Medicine (M.D.), and Master of Surgery (M.S.).

The student who desires to take the degree of M.B. of the London University has to pass the University Matriculation Examination, held thrice yearly in January, September, and June. The matriculation is accepted as a preliminary examination for graduation or qualification in medicine, provided the candidate has taken Latin as one of the optional subjects, and formerly no other examination exempted from the matriculation so far as degrees of the University were concerned. Recently, however, the Senate has seen fit to rule that several other examinations may be accepted instead of the usual matriculation.

After matriculating the student must attach himself to one or other of the various medical schools, and devote himself during his first year to mastering the subjects—chemistry, biology, and physics—required for his preliminary scientific or first professional examination. It will take him a full year to work up these subjects.

The student is at present admitted to the Intermediate examination, the subjects of which consist of anatomy, physiology, and pharmacology, two years after having passed Part I. of the Preliminary. Students who have passed the B.Sc. (Pass) Examination with physiology, or who have obtained honours at the B.Sc. Examination, having taken the Pass Examination in physiology in respect of their subsidiary subject, will not be required to pass the physiological portion of the Intermediate Examination for M.B. Time spent in working at the subjects of anatomy and physiology is not recognised by the University until the student has passed the Preliminary. His second and third years are thus spent in work in the dissecting room and physiological laboratory.

Having passed his "Inter.," the student begins clinical work. Two years of clinical study were formerly required, but now three years will be necessary, while two and a half years will be the minimum period required for the preliminary subjects. Thus the full course will in future occupy at least five and a half years from the time of matriculation. As a special war measure the regulations have been relaxed in certain ways in favour of those presenting themselves for their Final Examinations. The full details of these relaxations of the regulations are contained in a blue pamphlet which can be obtained from the University authorities.

The last examination is written, oral, and practical. The candidate is required to examine and report on selected cases, to do practical bacteriological work, and to be acquainted with the essentials of operative surgery, though actual operations on the dead subject are not demanded.

